# Mercury Vapour Long-Lasting Exposure: Lymphocyte Muscarinic Receptors as Neurochemical Markers of Accidental Intoxication

**DOI:** 10.1155/2016/9783876

**Published:** 2016-10-31

**Authors:** E. Roda, A. Giampreti, S. Vecchio, P. Apostoli, T. Coccini

**Affiliations:** ^1^Laboratory of Clinical & Experimental Toxicology and Poison Control Centre and National Toxicology Information Centre, Toxicology Unit, IRCCS Maugeri Foundation, Medical Institute of Pavia, Pavia, Italy; ^2^Department of Medical and Surgical Specialties, Radiological Sciences, and Public Health, Section of Public Health and Human Sciences, University of Brescia and Occupational Medicine, Hygiene, Toxicology and Prevention Unit, Civil Hospital of Brescia, Brescia, Italy

## Abstract

*Introduction.* Chronic poisoning may result in home setting after mercury (Hg) vapours inhalation from damaged devices. We report a chronic, nonoccupational Hg poisoning due to 10-year indoor exposure to mercury spillage.* Case Report.* A 72-year-old man with polyneuropathy of suspected toxic origin. At hospitalization, toxicological clinical evaluations confirmed the altered neurological picture documented across the last decade. Periodic blood and urine Hg levels (BHg, UHg) monitoring were performed from admission (*t*_0_), until 1 year later (*t*_2_), paralleled by blood neurochemical markers assessment, that is, lymphocytes muscarinic receptors (l-MRs). At *t*_0_: BHg and UHg were 27 and 1.4 microg/L, respectively (normal values: BHg 1–4.5; UHg 0.1–4.5), associated with l-MRs increase, 185.82 femtomoL/million lymphocytes (normal range: 8.0–16.0). At *t*_1_ (two days after DMSA-mobilization test), BHg weak reduction, paralleled by UHg 3.7-fold increase, was measured together with further l-MRs enhancement (205.43 femtomoL/million lymphocytes). At *t*_2_ (eight months after two cycles of DMSA chelating therapy ending), gradual improving of clinical manifestations was accompanied by progressive decrease of BHg and UHg (4.0 and 2.8 microg/L, resp.) and peripheral l-MRs neurochemical marker (24.89 femtomoL/million lymphocytes).* Conclusion.* l-MRs modulatory effect supports their use as peripheral neurochemical marker in Hg poisoning diagnosis and chelation therapy monitoring.

## 1. Introduction

Prolonged exposure to mercury (Hg) vapour may result in clinical pictures of chronic poisoning. This lasting intoxication, usually due to Hg vapour inhalation, is characterized by an initial flu-like syndrome, affecting at the outset the respiratory tract with symptoms of cough, sore throat, shortness of breath, and chest pain, then followed by signs affecting gastrointestinal, central, and peripheral nervous system, with a wide range of symptoms including fever, erythematous rash, itching, chills, gastrointestinal complaints, metallic taste, headache, and weakness. Anyway this “metal fume fever,” a syndrome commonly confused with a viral etiology, still remains a poorly understood syndromic picture [1].

Mercury poisoning is mainly described and reported in medical literature as a result of occupational exposure. Nonetheless, chronic exposure to Hg vapour is also possible in domestic/nonoccupational setting. Particularly, with the wide utilization of Hg in thermometers, sphygmomanometers, and barometers, mainly used at home and in hospitals as well as in schools, an accidental breakage of these devices may cause spillage of Hg droplets resulting in a chronic elemental mercury intoxication [1–3].

Additionally, indoor unintentional exposure may involve domestic appliances, such as the newest artificial lighting systems, for example, compact fluorescent lamps (CFLs), which are known to be energy-efficient compared to incandescent bulbs but contain milligram (mg) quantities of Hg. Particularly, international concerns have been raised regarding potential Hg vapour exposures following CFLs breakage, and various efforts have focused on managing this issue [4, 5].

Mercury used in consumer products is metallic Hg. Inhalation is usually the main route of concern because 80% of inhaled Hg is absorbed. After inhalation, elemental-Hg is readily absorbed through the alveolar membrane and transported by the blood to the brain and other target tissues. The susceptibility of central nervous system (CNS) to Hg is well established according to epidemiological and experimental investigations. Moreover, substantial evidences showed that cholinergic muscarinic system can be affected by* in vitro* and* in vivo* exposure to Hg [6]. Indeed literature data demonstrated that some environmental neurotoxic chemicals, other than Hg, may influence cholinergic muscarinic system by a variety of mechanisms. For example, organophosphates interact directly with receptor protein, acting either as agonist or as antagonist. Moreover, other agents may alter the receptors indirectly, either by changing the levels of endogenous neurotransmitter acetylcholine (as in the case of organophosphorus insecticides) or by damaging muscarinic receptor-bearing cells (e.g., trimethyltin) [7].

Concerning the Hg, because the body eliminates this metal slowly, cumulative exposure is the primary matter of concern, being the cause of a wide range of heavy health adverse effects [8].

Mercury chronic poisoning syndrome includes neuropsychiatry disturbances as well as peripheral neuropathy and renal involvement (presenting as proteinuria or tubulopathy). In particular, neurological symptoms may include decreased nerve impulse conduction, decreased motor skills (e.g., finger tapping, and hand-eye coordination), irritability, poor concentration, shyness, tremors (initially affecting the hands and sometimes spreading to other parts of the body), incoordination (e.g., difficulty walking), and short-term memory loss. The motor skill effects may be reversible, but short-term memory loss may be permanent [8]. Moreover, severe hypertension due to catecholamine excess was described in previous reports [2].

From a clinical point of view, misdiagnosis of Hg poisoning, often as a flu-like syndrome (at early onset) or as a psychological disorder (at later stages), is a common problem. Sometimes, before the correct diagnoses, patients worsen after returning to the place of contamination [8].

Because exposure to neurotoxicants, including Hg, may cause biochemical and molecular events indicating early-stage effects of exposure preceding the onset of overt disease, monitoring these early events may represent a valuable approach, employing neurotoxicity markers as useful tool for detecting subclinical disease states and initial detrimental changes associated with long term low-dose exposure to Hg, thus supporting the clinicians in making an early differential diagnosis.

We report a case of chronic, nonoccupational mercury poisoning due to 10-year prolonged Hg vapour exposure as a result of spillage from broken barometers at home, paralleled by related alteration in peripheral neurochemical parameters, that is, lymphocytes muscarinic receptors. Our laboratory data clearly supported the use of this peripheral biomarker as susceptible target for Hg neurotoxicity in human.

## 2. Case Presentation

A 72-year-old man (70 kg body-weight) presented to our Toxicology Unit (TU) with a 10-year past medical history of progressive neurological symptoms (Table 1), to investigate a polyneuropathy of suspected toxic origin. The patient's occupational history was negative for previous exposure to metals. A nuclear magnetic resonance (NMR) performed 5 years before was negative for brain lesions. The patient mentioned the presence of a big broken barometer at his home, maintained near heating source in his study-room during the last ten years.

Notably, in October 2010, after a private consultation with a Belgian neurologist in Anversa, abnormal enhanced BHg levels were determined, that is, 36 microg/L at first control. Thus, chelation therapy cycles with administration of 2,3-dimercapto-1-propanesulfonic (DMPS) acid were prescribed by the Belgian physician. This therapy scarcely diminished BHg levels (26.7 and 21 microg/L after the first and second chelation cycles, resp.), also failing to produce any healthy relief on neurological symptoms (i.e., postural instability and sensory-motor polyneuropathy lasting) (Table 1). We may suppose that therapy inefficacy could be related to underdose, incorrect administration, and scarce adherence to the therapy, although these hypotheses are speculative since the patient was not directly managed by our Toxicology Unit during DMPS treatment. Moreover, it has to be mentioned that DMPS administration may be associated with side effects, such as allergic reactions and lowering in blood pressure, which could have contributed to a decrease in an elderly patient's compliance.

Hence, the patient was hospitalized (June 2011) at our clinical Toxicology Unit of IRCCS Salvatore Maugeri Foundation (FSM), Scientific Institute of Pavia. Toxicological clinical evaluation confirmed the altered neurological picture documented across the last decade (Table 1), characterized by motor ataxia, postural instability, positive Romberg, increased motor tone, paresthesias, and sensory deficits in-touch at inferior limbs. Neurophysiological tests (e.g., motor-sensory electroneurography (ENeG) and vegetative nervous system compartment electroneurography) revealed a mild axonal sensory-motor polyneuropathy at superior and inferior limbs. Parallel neuropsychological evaluation (NPE) demonstrated a normal global cognitive functioning. A standard electroencephalogram (EEG) was read as within normal limits.

Standard laboratory investigations demonstrated mild anisopoikilocytosis, microcytosis, and anemia, consistent with typical heterozygous beta-thalassemic traits. Furthermore, specific haematochemical tests (Table 2), revealed an increased concentration (i.e., 67 microg/dL versus normal value: < 40 microg/dL) of erythrocyte zinc protoporphyrin (ZnPP) only. In accordance with previous clinical investigations [9, 10], we related this latter slightly altered parameter to the patient thalassemic phenotype, since the value measured at admission remained stable during all monitoring period, until eight months after the end of the complete chelation therapy.

Potential kidney effect was also investigated by evaluating standard renal function markers, that is, creatinine, uric acid, electrolytes, serum beta-2 microglobulin, and N-acetyl-beta-D-glucosaminidase, all resulting in healthy reference ranges, and clinical tests, for example, Giordano's sign, resulted bilaterally negative. Other laboratory findings fell in the considered normal range (data not shown).

### 2.1. Methods in Brief

We periodically monitored Hg concentrations in blood and urine (BHg and UHg) from admission to our TU (*t*_0_) until 1 year later (about 410 days) (*t*_2_), around eight months after the end of the complete chelation therapy. For the urinary Hg determination, 24-hour urine specimens were collected.

At admission (*t*_0_), even rare metals and trace elements (e.g., lead, zinc, magnesium, and manganese) were also measured. All the evaluated levels fell in the healthy normal ranges (data not shown).

Notably, two days after admission, a chelation challenge test with* meso*-2,3-dimercaptosuccinic acid (DMSA, Succicaptal®), reflecting the mobilizable and likely toxicologically active fraction of the Hg body burden, was performed (two equal oral doses every 8 hrs: 10 mg/kg/dose), strictly monitoring BHg and UHg.

#### 2.1.1. Mercury Determination

Mercury levels were determined by inductively coupled plasma-mass spectrometry (ICP-MS) while speciation analyses were conducted using HPLC-ICP-MS [11, 12].

Accuracy was checked by reference solutions (8AB occupational G-EQUAS for blood and urine). The detection limits and the coefficients of variability (CV%) for the different matrices were 0.1 microg/L and 5% for BHg and 0.05 microg/L and 4% for UHg, respectively.

#### 2.1.2. Blood Cells Isolation and Determination of Peripheral Neurochemical Markers, That Is, Muscarinic Receptors in Lymphocytes (l-MRs) and Monoamine Oxidase B in Platelets (p-MAO-B)

For l-MR and p-MAO-B determinations in lymphocytes and in platelets, respectively, blood samples were collected into EDTA containing tube and immediately processed to isolate lymphocytes for MR binding or platelets for MAO-B activity as previously described [13]. The p-MAO-B activity was determined radiochemically in duplicate samples as described by Coccini et al. using ^14^C-PEA as the substrate [14]. Specific activity was determined in the presence of pargyline hydrochloride. The enzyme activity was expressed as nanomoL/mg protein/h.

Muscarinic receptors were determined by binding assays using a single concentration (Kd) of the specific tritiated ligand antagonist ^[3H]^QNB for muscarinic receptors in lymphocytes [13]. The specific binding was measured in the presence or absence of atropine. Each sample was assayed in triplicate and data were expressed as femtomoL/10^6^ cells.

## 3. Results

**Figure 1:**
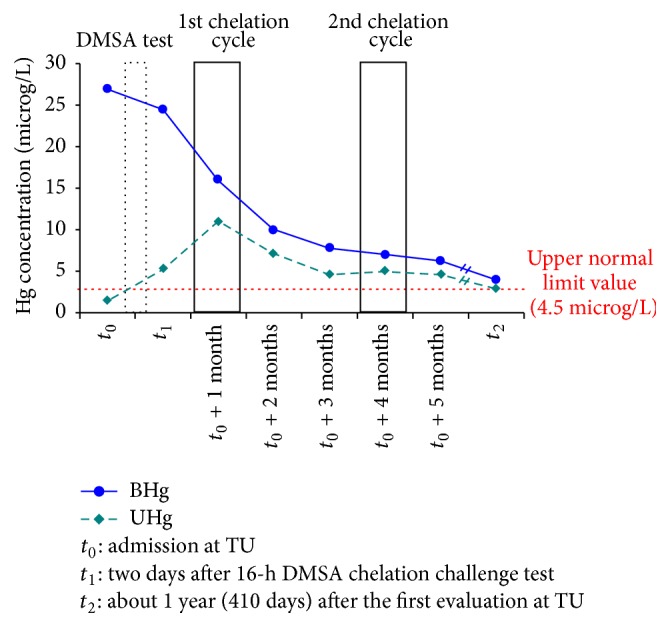
Hg concentration trends in different sampled matrices, that is, blood (BHg) and urine (UHg), throughout the entire biological monitoring 1-year period (from *t*_0_ to *t*_2_). The hatched bars indicate the 16-h chelation challenge test and the successive two DMSA cycles.

**Figure 2:**
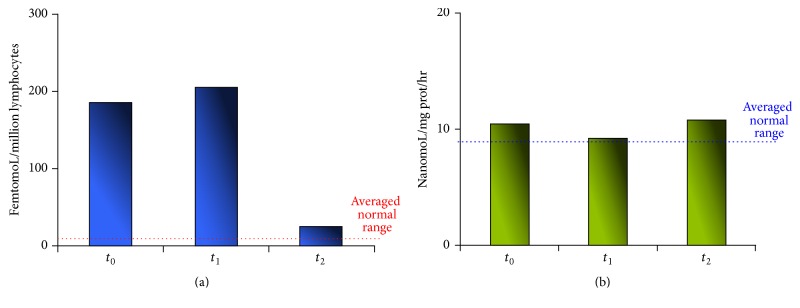
Measured neurochemical markers levels at admission (*t*_0_), two days after DMSA chelation challenge test (*t*_1_), and about 1 year (410 days) (*t*_2_) after the TU admission. (a) Lymphocytes muscarinic receptors (l-MRs). (b) Platelets monoamine oxidase B activity (pMAO-B).

The patient was evaluated and monitored both during the 7-day hospitalization at TU of FSM Pavia hospital and throughout the following 1-year period.

(I) *t*_0_ *: at admission to our Toxicology Unit*, BHg and UHg levels were 27 and 1.4 microg/L, respectively (normal values: BHg 1–4.5 microg/L; UHg 0.1–4.5 microg/L) (Figure 1). Parallelly, neurochemical markers, that is, muscarinic receptors in lymphocytes (l-MRs) and monoamine oxidase B in platelets (p-MAO-B), have been determined. The latter analyses demonstrated (i) a strong, significant increase in lymphocyte-MRs, that is, 185.82 femtomoL/million lymphocytes (normal range: 8.0–16.0), and (ii) a normal p-MAO-B activity of about 10.46 nanomoL/mg prot/hr (normal range: 7.0–11.0) (Figures 2(a) and 2(b)).
(II) *t*_1_ *: two days after the DMSA (Succicaptal) chelation challenge test*, (i) a weak reduction of BHg concentration was measured, paralleled by a 3.7-fold increase of UHg concentration. Specifically, the following BHg and UHg levels were measured: 24.5 and 5.2 microg/L, respectively (Figure 1). Elemental-Hg (before and after mobilization) and methyl-Hg (after mobilization) were evidenced by Hg speciation analysis in blood; specifically, methyl-Hg concentration was found to be about 1% of the total BHg; (ii) at the same time, neurochemical marker assessment was conducted, evidencing a further enhancement in l-MRs, that is, 205.43 femtomoL/million lymphocytes (normal range: 8.0–16.0), while p-MAO-B activity remained at physiological levels (9.22 nanomoL/mg prot/hr) (Figures 2(a) and 2(b)).
(III) * One week after admission*, based on anamnestic history, Hg levels, and data from investigation performed at TU (neurophysiological and neuropsychological evaluation together with haematochemical tests), the diagnosis of chronic Hg intoxication was established; thus a chelation therapy was prescribed. Specifically, the patient received oral administration of DMSA (two cycles: 1st cycle: 2400 mg/die for 5 days, followed by 2nd cycle: 1600 mg/die for 14 days, about three months later). The patient did not report any side-effect during the administration of the chelating agent. During this period, the measurements of the BHg and UHg concentrations have been repeated for five consecutive months, showing a decreasing trend (Figure 1). After the initiation of DMSA therapy, a gradual improving in clinical manifestations associated with a progressive reduction of BHg and UHg levels was observed (Figure 1).
(IV) *t*_2_ *: notably, about 1 year (410 days) after the first evaluation at TU admission*, a further, last evaluation was performed, assessing both Hg levels and neurochemical markers. These latter determinations demonstrated (i) a marked reduction of l-MRs (24.89 femtomoL/million lymphocytes), showing an evident tendency to normalize (normal range: 8.0–16.0), and (ii) an unaltered p-MAO-B activity (10.74 nanomoL/mg prot/hr), displaying normal, unchanged value, compared to those measured at previous time-points (*t*_0_ and *t*_1_) (Figures 2(a) and 2(b)).

Accordingly, BHg and UHg concentrations were settled on normal reference value, showing negligible difference with those determined eight months before, at the end of the chelation therapy (*t*_0_ + 5 months) (Figure 1).

Importantly, it should be highlighted that parallel neurophysiological evaluation demonstrated a complete remission of the detrimental neurological symptoms.

## 4. Discussion

Evaluating the effects of exposure to neurotoxicants is extremely difficult in human investigations. The Hg exposure described in the present case report is quite different than that occurring in classical occupational setting. This accidental Hg vapour long-lasting exposure is hardly quantifiable, characterized by inhomogeneous, unlikely predictable indoor concentrations. If it is widely accepted that repeated and regular chronic Hg exposure (e.g., in occupational setting) causes increases of both urine and blood Hg levels, on the other hand, so far, the outcome of biomarkers expected in a peculiar environmental exposure scenario like the one described in our case report is not clearly documented. Specifically, the patient exposure to Hg in the study-room was irregular, lasting an amount of time different day by day, with Hg air concentration unpredictable and probably dissimilar in the different areas of the room. Our findings demonstrated a high BHg value already at admission, paralleled by a normal UHg level. Particularly, even though a physiological kidney function was observed (as indicated by normal renal parameters as well as clinical evaluation), unexpectedly we did not determine an enhanced Hg renal excretion at admission. We can hypothesize that Hg remained deposited in some tissues (particularly in nervous system), as demonstrated by the detrimental neurological symptoms. Moreover it is possible that this “irregular” exposure led a Hg accumulation in patient sufficiently to induce neurotoxicity without causing an evident Hg increase in urine. In this case report, Hg exposure may be considered as the consequence of repeated short exposures, some hours a day only and even not every day: this exposure pattern could justify the Hg increase in blood (as indicator of occurring exposure) associated with low Hg urine levels resulting from repeated but “irregular” and spotted exposures. In support of this hypothesis, the DMSA chelation challenge test induced a 3.7-fold increase of UHg concentration, associated with a weak reduction of BHg concentration, thus demonstrating the mobilization of Hg levels from tissues and the atypical chronic accumulation mirrored by the atypical Hg urinary levels at *t*_0_.

In this respect, the present study applying a complementary approach, which correlates specific exposure parameters (i.e., analytical data) and indicators of neural cell function, in peripheral blood cells, supported and properly addressed a differential diagnosis, thus representing a promising strategy to be used in clinical setting.

One characteristic of the neurochemical parameters presently investigated, that is, muscarinic receptors (MRs) and monoamine oxidase activity type-B (MAO-B), is that they are also expressed in easily accessible matrices, for example, blood components, such as lymphocytes and platelets.

Although novel noninvasive radiological imaging techniques such as NMR, PET, and SPECT (magnetic resonance, positron emission tomography, and single-photon emission computed tomography, resp.) may allow directly estimating MRs and MAO activity in living human brain [15], these methods are expensive in terms of cost and their widespread application to neurotoxicological investigations can not be proposed on a large scale, thus supporting the need to employ the above-mentioned peripheral neurochemical parameters, to investigate the status of homologous CNS markers [16–18].

Even though the use of biochemical markers in neurotoxicology is particularly challenging due to (i) the complexity of CNS functions, (ii) the multistage nature of neurotoxic events, and (iii) the inaccessibility of target tissue, in recent years great effort has been devoted to develop and validate new surrogate parameters in peripheral tissues easily and ethically obtained in humans, reflecting the same parameters in nerve tissue.

Specifically, peripheral blood lymphocytes are considered the main tool to explore cholinergic function, as also supported by human studies demonstrating similar immunoblotting patterns both in lymphocyte and in striatum membranes [19]. Further experimental evidence showed that MRs binding can be similarly modulated by cholinergic agonists and antagonists, in both lymphocytes and brain tissue [20].

For these intriguing peculiarities, in our previous studies, intended to validate their use as peripheral surrogate markers in experimental controlled conditions, MRs have been investigated as biomarkers of neurotoxicity in animals exposed to environmental chemicals, demonstrating to reflect analogous receptor changes occurring in rat brain after repeated MeHg exposure during adult age as well as during development [6, 21].

Additionally, peripheral MRs have been clinically applied as predictors of pharmacological response in psychotropic drugs-treated subjects, as well as to investigate the role of neurochemical disturbances in affective disorders and neurological diseases (e.g., Alzheimer's, Parkinson's and Meniere's diseases, and Gilles de la Tourette syndrome), clearly demonstrating significative MRs binding alterations [16, 17, 19].

Moreover, our previous investigation showed a significant level reduction of surrogate peripheral markers of cholinergic and monoaminergic neurotransmissions in attention deficit hyperactivity disorder (ADHD) children. Particularly, a relationship has been demonstrated between l-MR binding levels and specific ADHD symptoms such as inattention and ODD in unmedicated subjects [22].

With regard to the other measured peripheral marker, that is, platelet MAO-B (p-MAO-B), it is the sole type in human platelets and the primary type in the human brain (80–95% of total MAO), playing a pivotal role in the catabolism of various neuroactive and vasoactive amines, that is, neurotransmitters (including dopamine), being located in CNS, as well as many peripheral tissues.

The amino acid sequences of MAO-B in both platelets and brain are identical and the biochemical and pharmacological characteristics of this isoenzyme are also similar in the two tissues. For these reasons, p-MAO-B activity was proposed as a predictive peripheral marker of various psychopathologies [23], neurodegenerative diseases [24], and CNS neurotoxic alterations [21]; further, alterations in MAO levels have been implicated in the pathogenesis of psychiatric disorders. As such, decreased platelet MAO-B activity was found in children with ADHD [22, 25]. Altered p-MAO-B has been also suggested as a biomarker of alcohol dependence or alcohol consumption [14, 26].

With regard to neurotoxic compounds, this platelet enzyme has also been applied as peripheral biomarker of monoamine neurotransmission in patients exposed to neurotoxicants such as styrene [27] or environmental Hg [28].

In summary, positive support in the use of the neurochemical markers clearly emerges from a bulk of human literature data providing typical alteration of these parameters when used (i) as peripheral indicators of the brain neurochemistry changes associated with neuropsychiatric disorders and drug dependence or (ii) as predictors of environmental neurotoxicants exposure.

Moreover, our previous papers delineate the relevant contribution of* in vivo* (animal and human) researches to identifying specific molecular CNS targets of neurotoxicants which can be applied as accessible tools to use in environmental medicine as well as in clinical setting for assessing and monitoring specific exposure scenarios [13, 21].

In this view, integrated investigation approach using peripheral neurochemical markers in combination with clinical neurological evaluation and analytical data (contextually considered with patient's history) could represent a valuable methodological strategy by which human neurotoxicity assessment may become more focused, particularly in chronic exposures.

The study clearly shows an evident association among Hg exposure levels in biological specimens, blood cholinergic markers, and clinical manifestation trend. Specifically, high/low BHg and UHg levels were accompanied by high/low MRs in lymphocytes as well as severe/slight neurological symptoms.

This is the first documented case in human of a valuable application of a specific neurochemical biomarker enabling the clinicians to support specific early differential diagnosis (i.e., Hg poisoning) and to monitor the chelation therapy efficacy.

## Figures and Tables

**Table 1:** Clinical and neurological events/evaluations during the decade (2000–2011) before admission to our Toxicology Unit.

2000–2005	Neurological alterations onset and progression: postural instability during deambulation, associated with paresthesias and hypoesthesia at anterolateral surface of thighs; in 2003, after prostatectomy, hypoesthesia extended to tailbone area, paralleled by pain worsening at inferior limbs			
	*Medical neurological division/consultant*	*Lab test/analyses*	*Symptoms/diagnosis*	*Therapy*

*2006* (hospitalized)	San Raffaele Hospital, Cefalù, Italy	(i) Supra-aortic trunks and inferior limbs color-Doppler (CD) (ii) Electromyography (EMG) and motor evoked potentials (MEP) (iii) Encephalic and spinal column magnetic resonance imaging (MRI)	(i) Sensory-motor polyneuropathy at inferior limbs (ii) Hyperintense Punctate frontobilateral subcortical foci of gliosis (iii) Spinal disc herniations and lumbar discal bulging (iv) Vertebral hemangiomas in some metameric segments at dorsal and lumbar levels	Gabapentin: lack of detailed pharmacological plan documentation Therapeutic drug treatment self-suspended by the patient

*2007* (hospitalized)	San Raffaele Foundation Scientific Institute Hospital, Neurology, Clinical Neurophysiology and Neurorehabilitation, Milan, Italy	(i) Electromyography (EMG), motor evoked potentials (MEP), somatosensory evoked potential (SSEP) monitoring (ii) Sural nerve biopsy (iii) Haematochemical tests (iv) Abdominal ultrasound	(i) Sensory-motor polyneuropathy (ii) Alteration in peripheral and radiculomedullary somatosensory conduction (iii) Motor and sensory nerve conduction abnormalities (iv) Axonal damage (v) Monoclonal gammopathy of the IgG lambda type (vi) Gallbladder adenomyoma *Diagnosis* Cordonal syndrome and idiopathic peripheral neuropathy (unknown etiology)	Lack of documentation

*2008* (January–March)	—	Self-evaluation	(i) Postural instability progression	Self-administration of betamethasone (2 mg/die)

*2008* (September, hospitalized)	Neurology and Neurophysiology, Policlinico *P. Giaccone* Hospital, Palermo	(i) Motor evoked potentials (MEP) and somatosensory evoked potential (SSEP) monitoring (ii) Haematochemical tests (including Antinuclear Antibody (ANA) test)	(i) Postural instability progression (ii) Motor and somatosensory conduction abnormalities (iii) ANA test positivity: 1 : 80 *Diagnosis* “Sensorymotor neuropathy of undetermined cause and spondylogenic myelopathy” paralleled by monoclonal gammopathy of undetermined significance (MGUS)	Lack of documentation

*2009* (clinical consultation)	*Carlo Besta* Neurological Institute, Milan	(i) Neurological evaluation	Lack of documentation	Dexamethasone 25 mg/die

*2010–January 2011*	Private consultation with a neurologist, Anversa, Belgium	(i) Blood mercury levels determination	(i) Postural instability progression (ii) Sensory-motor polyneuropathy (iii) Abnormal enhanced blood mercury levels (i.e., 36 microg/L at first control and 26.7 and 21 microg/L after the first and second chelation cycles, resp.)	Chelation therapy cycles with i.v. administration of 2,3-dimercapto-1-propanesulfonic (DMPS) acid

**Table 2:** Laboratory parameters evaluated for the clinical assessment of the patient on admission at Toxicology Unit of IRCCS Salvatore Maugeri Foundation.

Parameters	Matrices	Value	Reference normal levels
Coproporphyrin I	Urine/24 h	7.65 microg/24 h	<25
Coproporphyrin III	Urine/24 h	59.3 microg/24 h	<75
Uroporphyrin	Urine/24 h	12.7 microg/24 h	<25
*δ*-Aminolevulinic Acid	Urine/24 h	2.1 mg/24 h	0.25–6.4
Porphobilinogen	Urine/24 h	1.22 mg/24 h	0.1–1.7
N-Acetyl-beta-D-glucosaminidase	Urine/24 h	5.14 IU/L	0.3–12
*δ*-Aminolevulinic acid dehydratase	Blood	75 U/mL	>20
Zinc protoporphyrin	Blood	67 microg/dL^*∗*^	<40

^*∗*^Out of normal levels.

## References

[1] Cicek-Senturk G., Altay F. A., Ulu-Kilic A., Gurbuz Y., Tutuncu E., Sencan I. (2014). Acute mercury poisoning presenting as fever of unknown origin in an adult woman: a case report. *Journal of Medical Case Reports*.

[2] Koyun M., Akman S., Güven A. G. (2004). Mercury intoxication resulting from school barometers in three unrelated adolescents. *European Journal of Pediatrics*.

[3] Langley R., Hirsch A., Mcdanie J. (2014). Elemental mercury spill in school bus and residence-North Carolina, 2013. *Morbidity and Mortality Weekly Report*.

[4] Lim S.-R., Kang D., Ogunseitan O. A., Schoenung J. M. (2013). Potential environmental impacts from the metals in incandescent, compact fluorescent lamp (CFL), and light-emitting diode (LED) bulbs. *Environmental Science and Technology*.

[5] Nance P., Patterson J., Willis A., Foronda N., Dourson M. (2012). Human health risks from mercury exposure from broken compact fluorescent lamps (CFLs). *Regulatory Toxicology and Pharmacology*.

[6] Coccini T., Randine G., Castoldi A. F., Acerbi D., Manzo L. (2007). Methylmercury interaction with lymphocyte cholinergic muscarinic receptors in developing rats. *Environmental Research*.

[7] Costa L. G., Lester D. S., Slikker W., Lazarovici P. (2003). Cholinergic muscarinic receptors as target for neurotoxicity. *Site-Selective Neurotoxicity*.

[8] Baughman T. A. (2006). Elemental mercury spills. *Environmental Health Perspectives*.

[9] Estévez N. S., Herruer M. H., Jansen R., Bergkamp F. J. M., Gorgels J. P. M. C. (2009). Diagnostic value of zinc protoporphyrin in a screening strategy for *α*-thalassemia. *European Journal of Haematology*.

[10] Graham E. A., Felgenhauer J., Detter J. C., Labbe R. F. (1996). Elevated zinc protoporphyrin associated with thalassemia trait and hemoglobin E. *Journal of Pediatrics*.

[11] Apostoli P., De Palma G., Catalani S., Bortolotti F., Tagliaro F. (2009). Multielemental analysis of tissues from Cangrande della Scala, Prince of Verona, in the 14th century. *Journal of Analytical Toxicology*.

[12] Apostoli P., Catalani S., Sigel A., Sigel H., Sigel R. K. O. (2011). Metal ions affecting reproduction and development. *Metal Ions in Toxicology: Effects, Interactions, Interdependencies: Metal Ions in Life Sciences*.

[13] Coccini T., Randine G., Castoldi A. F., Balloni L., Baiardi P., Manzo L. (2005). Lymphocyte muscarinic receptors and platelet monoamine oxidase-B as biomarkers of CNS function. Effects of age and gender in healthy humans. *Environmental Toxicology and Pharmacology*.

[14] Coccini T., Castoldi A. F., Gandini C. (2002). Platelet monoamine oxidase B activity as a state marker for alcolism: trend over time during withdrawal and influence of smoking and gender. *Alcohol and Alcoholism*.

[15] Fowler J. S., Logan J., Volkow N. D., Wang G.-J., MacGregor R. R., Ding Y.-S. (2002). Monoamine oxidase: radiotracer development and human studies. *Methods*.

[16] Rabey J. M., Lewis A., Graff E., Korczyn A. D. (1992). Decreased (^3^H) quinuclidinyl benzilate binding to lymphocytes in Gilles de la Tourette syndrome. *Biological Psychiatry*.

[17] Masuyama K., Uno K., Minoda R., Eura M., Samejima Y., Ishikawa T. (1996). Muscarinic acetylcholine receptors on human lymphocytes in patients with Meniere's disease. *Acta Oto-Laryngologica*.

[18] Farren C. K., Tipton K. F. (1999). Trait markers for alcoholism: clinical utility. *Alcohol and Alcoholism*.

[19] Tayebati S. K., El-Assouad D., Ricci A., Amenta F. (2002). Immunochemical and immunocytochemical characterization of cholinergic markers in human peripheral blood lymphocytes. *Journal of Neuroimmunology*.

[20] Costa L. G., Kaylor G., Murphy S. D. (1990). *In vitro* and *in vivo* modulation of cholinergic muscarinic receptors in rat lymphocytes and brain by cholinergic agents. *International Journal of Immunopharmacology*.

[21] Coccini T., Randine G., Castoldi A. F. (2006). Effects of developmental co-exposure to methylmercury and 2,2′,4,4′,5,5′-hexachlorobiphenyl (PCB153) on cholinergic muscarinic receptors in rat brain. *NeuroToxicology*.

[22] Coccini T., Crevani A., Rossi G. (2009). Reduced platelet monoamine oxidase type B activity and lymphocyte muscarinic receptor binding in unmedicated children with attention deficit hyperactivity disorder. *Biomarkers*.

[23] Oreland L., Hallman J., Damberg M. (2004). Platelet MAO and personality—function and dysfunction. *Current Medicinal Chemistry*.

[24] Zhou G., Miura Y., Shoji H., Yamada S., Matsuishi T. (2001). Platelet monoamine oxidase B and plasma *β*-phenylethylamine in Parkinson's disease. *Journal of Neurology Neurosurgery and Psychiatry*.

[25] Nedic G., Pivac N., Hercigonja D. K., Jovancevic M., Curkovic K. D., Muck-Seler D. (2010). Platelet monoamine oxidase activity in children with attention-deficit/hyperactivity disorder. *Psychiatry Research*.

[26] Snell L. D., Ramchandani V. A., Saba L. (2012). The biometric measurement of alcohol consumption. *Alcoholism: Clinical and Experimental Research*.

[27] Checkoway H., Echeverria D., Moon J.-D., Heyer N., Costa L. G. (1994). Platelet monoamine oxidase B activity in workers exposed to styrene. *International Archives of Occupational and Environmental Health*.

[28] Stamler C. J., Abdelouahab N., Vanier C., Mergler D., Chan H. M. (2006). Relationship between platelet monoamine oxidase-B (MAO-B) activity and mercury exposure in fish consumers from the Lake St. Pierre region of Que., Canada. *NeuroToxicology*.

